# The Soot that Falls from Chimneys

**DOI:** 10.3201/eid1609.AC1609

**Published:** 2010-09

**Authors:** Polyxeni Potter

**Affiliations:** Author affiliation: Centers for Disease Control And Prevention, Atlanta, Georgia, USA

**Keywords:** Art science connection, emerging infectious diseases, respiratory infections, art and medicine, Thomas Hart Benton, regionalism, legionellosis, Interior of a Farmhouse, The Soot that Falls from Chimneys, about the cover

**Figure:**
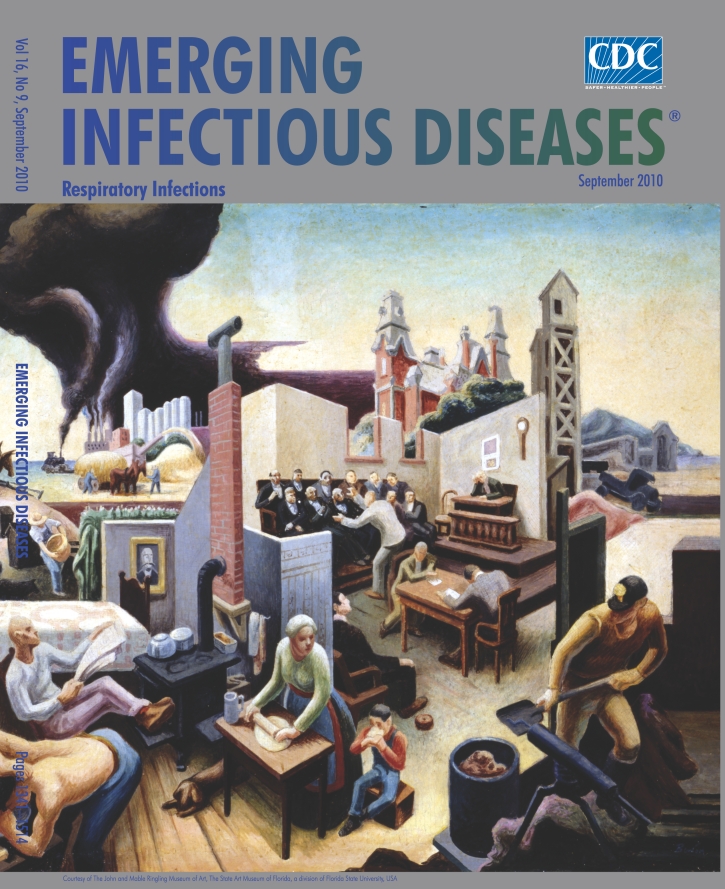
**Thomas Hart Benton (1889–1975), *Interior of a Farm House* (1936)** Tempera on board (45.7 cm × 76.2 cm), Courtesy of The John and Mable Ringling Museum of Art, The State Art Museum of Florida, a division of Florida State University, USA

“The best damned painter in America,” is how Harry S. Truman described Thomas Hart Benton, his choice to create a mural for the Truman Library in Independence, Missouri. The President’s fellow Missourian lived in Kansas City at the time, his artistic career in a slump, though his reputation as a fine muralist still intact. “I picked him,” Mr. Truman told the crowd at the mural’s dedication, “because he was the best, and this is the finest work by the best.”

During work on the Library mural, the President noted that he got along with the painter, even though, he joked, “That’s hard for anyone to do.” A gifted musician, writer, and prolific lithographer, Benton was also direct, impatient, radical, and often tactless. He hated museums, professing that art works belonged in clubs and barrooms, “Anywhere anybody had time to look at ’em.” In his autobiography, he mused, “A few people have, at times, expressed a belief that I was not the most desirable kind of fellow to have around. But, all in all, my differences with the home folks, when looked at in perspective, have not amounted to much.”

Born in a fiercely political family, the artist was named after his uncle Thomas Hart Benton, the first and longest serving U.S. Senator of Missouri. His father, a populist, was a member of the U.S. House of Representatives. Benton bucked family tradition and entered the Art Institute of Chicago, in 1907, aspiring to become a cartoonist. His longtime love of painting affirmed in the fine arts environment, he traveled to Paris, where he attended the Académie Julien and Académie Colarossi. He met Mexican muralist Diego Rivera and studied the masters at the Louvre, among them El Greco, whose exaggerated forms found their way into his mature style.

Back in New York for the lack of funds, he explored the local scene and such prevailing styles as impressionism and synchromism, especially in the work of Stanton MacDonald-Wright, who became his friend. His career took a different turn when he joined the Navy. “The most important thing, so far, I had ever done for myself as an artist.” This work, which involved extensive documentary drafts and drawing, had an enduring effect on his style. “When I came out of the Navy after the First World War, I made up my mind that I wasn’t going to be just a studio painter, a pattern maker in the fashion then dominating the art world―as it still does. I began to think of returning to the painting of subjects, subjects with meanings, which people in general might be interested in.”

Though by this time well-versed in modernism, Benton turned against it unable to embrace its “colored cubes and classic attenuations,” rejecting it as “divorced from the common ways of the day.” He moved toward naturalistic and representational work focused on the American scene. He taught at the Art Students League, instructing many who went on to become famous in their own right, not the least of them abstract expressionism icon Jackson Pollock. “Even after I had castigated his innovations and he had replied by saying I had been of value to him only as someone to react against, he kept in personal touch with me ….”

Benton’s style, a blend of modern and academic elements, came to be known as regionalism. Others in this movement were Grant Wood, most famous for his *American Gothic*, and John Steuart Curry, who painted life in his native Kansas. Many criticized their choice of the local over the cosmopolitan, but the regionalists, particularly Benton, struggled with the notion of an authentic American voice long before New York became an art center. “You just can’t think of art in terms of progress,” Benton explained in an interview, “It is not progressive. It is just different from age to age. One age gets used to a certain kind of art form and thinks that is better, but the next age will deny that thought and go back to some older form. So I wouldn’t compare the animal paintings of the cavemen with those of our times or any other times …. We were as good, as artists, when we began our history as we are now―sometimes better.”

The 1930s, a decade of unprecedented economic hardship in the United States, witnessed renewed interest in history reflected in all aspects of culture. Murals, among them *The Social History of Missouri* in Jefferson City, which Benton considered his masterpiece, were part of this resurgence, recording as they were milestones in the country’s development. Benton captured his era’s transformation from rural and agrarian to urban and industrial. He painted the growth of business and technology, and the consequent changes in the lives of the common people, in paintings of steamboats and trains, factories, logging and mining operations, offices and farmhouses. “History was not a scholarly study for me but a drama.” He was innovative, bold, outspoken, and unafraid of controversy, allowing myth to blend with observation, casting ordinary people as heroes and pioneers. “I wasn’t so much interested in famous characters as I was in Missouri and the ordinary run of Missourians that I’d known in my life.”

Benton made a habit of gallivanting around the countryside, meeting people and sketching them and their surroundings. Later he would lay out his designs from these pencil sketches, using pen and ink to define and preserve them. These and three-dimensional clay models he created served as prototypes for oil and tempera studies and for larger compositions. While many critics objected to his subjects, bold colors, manipulated forms, or muscular style, few found fault with his compositional and architectural skills. He had a talent for incorporating multiple themes in limited space and still maintaining cohesiveness.

*Interior of a Farmhouse* on this month’s cover offers a glimpse of the brilliant color, energy, and movement that characterize Benton’s art and the complexity and richness of his murals. The title understates this intricate composition. The farmhouse at center stage anchors a community of scenes connected by a fence here, a doorway there, an angle, a partial wall, and contains his favorite people: workers doing what they do in the kitchen, the barn, the fields, at rest. On the periphery, steamboat navigation and the wheels of industry are rolling, their ubiquitous smokestacks belching above the Missouri River. Court is in progress; a worker reads the daily news; another washes up; animals wander in and outdoors. The painter reviews American industry in the 1930s, which pulsates, as if it were a live, breathing organism itself.

The values of honest living and hard labor, at the heart of Benton’s work, went hand in hand with the belief that harmony between humans and nature resided on the farm, the interior of which in this painting is not altogether filled with agrarian bliss. Despite the energy emanating from the vibrant community, there are tensions, political and ecologic undertones, part and parcel of industrialization. Benton the social historian sensed the dark side of factories and increased transportation, which he noted in palpable terms, a cloud so menacing against the pristine horizon it unfolded half way across the painting.

“The yellow smoke that rubs its muzzle on the window-panes/…Let fall upon its back the soot that falls from chimneys/,” Benton’s fellow Missourian T.S. Eliot wrote prophetically in “The Love Song of J. Alfred Prufrock.” As they settle, the dark plumes from smokestacks, a fixture in the artist’s work signaling the machine’s intrusion, cause havoc in the farmhouse. “The harmony man had with his environment has broken down,” he wrote. “Now men build and operate machines they don’t understand and whose inner workings they can’t even see.”

Choked by industrial and other pollution, we have come to resemble Benton’s farmhouse, an organism under stress, because “man doesn’t escape his environment.” The human lung, at the center of the body’s complex internal operations is also affected by external factors in the environment: pollution, infectious agents, allergens. These factors, along with causing many other local and global adverse effects, complicate and aggravate a host of respiratory problems from rhinovirus infection, influenza, and pneumonia, to pneumococcal disease, tuberculosis, and legionellosis, now found to spread around the community from the water tank of a paving machine. Once again, the farmhouse is threatened by what’s lurking in “The yellow fog that rubs its back upon the window-panes.”
